# Teachers' Growth Mindset and Work Engagement in the Chinese Educational Context: Well-Being and Perseverance of Effort as Mediators

**DOI:** 10.3389/fpsyg.2019.00839

**Published:** 2019-04-18

**Authors:** Guang Zeng, Xinjie Chen, Hoi Yan Cheung, Kaiping Peng

**Affiliations:** ^1^Department of Psychology, Tsinghua University, Beijing, China; ^2^Graduate School of Education, Stanford University, Stanford, CA, United States; ^3^Faculty of Education, University of Macau, Macau, China; ^4^Department Psychology, Tsinghua University, Beijing, China

**Keywords:** teachers, growth mindset, work engagement, well-being, perseverance, Chinese, effort, positive psychology

## Abstract

The current study investigated the relationships among growth mindset, work engagement, perseverance of effort and well-being for secondary school teachers in the Chinese educational context. We adopted Growth Mindset Inventory, Utrecht Work Engagement Scale (UWES), Grit Scale (Perseverance subscale), and PERMA profiles that measure five dimensions of well-being. Participants included 472 secondary school teachers from 10 secondary schools in central China. Correlation analysis showed that growth mindset, well-being, and perseverance of effort could all predict work engagement. Moreover, the structural equation model and mediation analysis further suggested that well-being and perseverance of effort could partially mediate the relationship between growth mindset and work engagement. This study permitted to advance our knowledge about the relationship between growth mindset and work engagement, which should be considered for future teaching practices and teacher development.

## Introduction

Growth mindsets, also called implicit theories, are defined as core assumptions about the malleability of personal qualities (Dweck and Leggett, 1988; Dweck et al., 1995; Molden and Dweck, 2006; Yeager and Dweck, 2012). People with a growth mindset (an incremental theory) hold the beliefs that intellect, personality, and ability as something that can be grown or developed over time. People with a fixed mindset (an entity theory) hold the belief that these basic human quality are static and unchangeable (Dweck, 2009; Yeager and Dweck, 2012). People who believed that intelligence as a malleable quality (as opposed to an unchangeable, fixed entity) demonstrated stronger learning goals, more positive beliefs about effort, and engaged in more effort-based strategies, such as working harder and spending more time on the task (Blackwell et al., 2007). When individuals set learning as their goal, they focus on the meaning behind what they have to learn, and they also try to make improvements for one's own sake (Abrami and McWhaw, 2001). On one hand, fixed mindset people emphasize on performance goals, such as looking smart and proving their own ability. On the other hand, growth mindset people emphasize on learning goals, such as becoming smart and improving their abilities (Dweck and Leggett, 1988; Dweck, 2000). Students with a growth mindset were more resilient in face of difficulties (Dweck, 2010). Growth mindset students are found to interpret human behaviors in terms of context-sensitive psychological process, whereas fixed mindset people emphasize deep-seated, cross-situational traits as crucial causes of behaviors (Chiu et al., 1997; Molden and Dweck, 2006). Additionally, fixed mindset individuals are more inclined to stereotyping (Levy et al., 1998; Rydell et al., 2007).

This study focuses on teachers' growth mindsets and work engagement. The following paragraphs investigated previous studies on work engagement and its relationship with growth mindset of teachers.

## Work Engagement

In order to prosper and develop in today's continuously changing environment, organizations need engaged employees. The concept of work engagement emerged from burnout research, aiming to cover the entire spectrum running from employee burnout (ill-being) to their well-being. Work engagement is defined as a positive, fulfilling, work-related state of mind that is characterized by vigor, dedication, and absorption (Schaufeli et al., 2002a,b). *Vigor* is represented by high levels of energy and mental resilience during working. *Dedication* means to be actively involved in one's work and to experience a sense of significance, enthusiasm, and challenge. *Absorption* is defined as being fully concentrated and happily engrossed in one's work, whereby time passes quickly (Schaufeli and Bakker, 2004). Work engagement has been frequently studied through the job demands-resources model developed by Bakker and Demerouti (2007). The model describes that in every job or career, there are specific risk factors, and these risk factors are classified in the categories of job demands and job resources.

There are many advantages to people who have high levels of work engagement. For example, Rich et al. (2010) found that it mediated between antecedents (value congruence, perceived organizational support, and core self-evaluations) and effects (task performance and organizational citizenship behavior). Roberts and Davenport (2002) listed out the advantages of having high work engagement, such as higher motivation and being more productive in their jobs. Teachers' work engagement was negatively related to job burnout and intention to quit (Hakanen et al., 2006; Høigaard et al., 2012). Moreover, Schaufeli and Bakker (2004) found that burnout and engagement were negatively related.

## Teachers' Growth Mindset and Engagement

Growth mindset has been discussed and applied to the educational system. Research demonstrated that teachers' implicit theories of ability will impact their behaviors in the classroom, including their instructional approaches (Swann and Snyder, 1980), their sense of self-efficacy (Strosher, 2003), and how they view initial performance over time (Plaks et al., 2001). Furthermore, teachers' implicit beliefs of intelligence largely influence their own behaviors and interactions with students (Georgiou et al., 2002; Rissanen et al., 2019). Besides, teachers play critical roles in influencing the students' beliefs about their own ability and communicate with them in subtle ways (Rattan et al., 2012; Schmidt et al., 2015). For example, growth mindset teachers encouraged students to try harder because they believed they could do better. Teachers can also shape students' views of their own abilities and further motivate them toward achievement in subtle ways of language use (Cimpian et al., 2007; Schmidt et al., 2015). Specifically, teachers with a fixed mindset praise their students' basic attributes more often (Jonsson and Beach, 2012), which exert negative impacts on students' learning motivation and perseverance of effort (Mueller and Dweck, 1998). Kärk-käinen and Räty (2010) found that children's interpersonal and intrapersonal perceptions of their potential for improvement tended to be related to their teachers' perceptions. Shim et al. (2013) confirmed that, although the effect of teachers' mindset about students' intelligence was meager, a significant connection between teachers' mindset and classroom performance structure was found to have an interactive effect. However, up-to-date, research is mainly focused on how teachers' mindset influences students' mindset, learning motivation, and academic achievement. There is little research on how teachers' mindset influences their work engagement, well-being, and perseverance in the educational settings.

Research demonstrated that changing students from a fixed mindset to a growth mindset exerted positive impact on their academic engagement (Aronson et al., 2002), academic performance (Good et al., 2003; Blackwell et al., 2007), and resilience toward setbacks (Blackwell et al., 2007; Burnette et al., 2013; Zeng et al., 2016). Growth mindset students interpret their academic challenges as chances to improve their ability and sharpen their learning skills, which contributes to their resilience and engagement on schoolwork, for both high and low achieving students (Blackwell et al., 2007; Nussbaum and Dweck, 2008). Theoretical speaking, an incremental theory of intelligence leads to positive effort, beliefs, and learning goals, which in turn leads to fewer ability-based, helpless attributions but more positive strategies. This leads to enhancement of engagement and performance (Blackwell et al., 2007). However, there is no direct evidence suggesting that growth mindset could lead to the enhancement of work engagement, in particular for teachers in the Chinese educational context. To fill this gap, the current study is to examine whether and how the growth mindset of teachers could influence their work engagement at school.

## Well-Being and Perseverance of Effort as Mediators

### Well-Being as Mediator

Previous studies showed that growth mindset could lead to well-being (e.g., Zeng et al., 2016; Mouratidis et al., 2017; Whittington et al., 2017). People with a fixed mindset are likely to select activities that they think they are capable of doing, so that their self-esteem can be reassured. They try to avoid any challenges that may put their intelligence at risk, which makes them vulnerable to setbacks (Whittington et al., 2017). When they face difficulties or failure, they would easily develop a helplessness response and would then interpret their whole self-identities negatively. In contrast, people with a growth mindset especially enjoy challenges and would select activities that could help them improve. When they face difficulties and failure, they apply the mastery approach by focusing on what went wrong and why so that they can improve in the future. People with the two different mindsets respond to setbacks and failure differently and thus leading them to have different degrees of happiness and well-being (Whittington et al., 2017). Therefore, mindset could affect students' well-being, such as subjective vitality and feelings of depression (Mouratidis et al., 2017). Zeng et al. (2016) showed a similar positive relationship between growth mindset and well-being in which different mindsets make individuals perceive things in different ways and that “growth mindset promotes resilience while fixed one does not” (p. 2).

Previous studies indicated that well-being and work engagement is positively associated, and they impacted each other (Shimazu and Schaufeli, 2009; Shimazu et al., 2012; Upadyaya and Salmela-Aro, 2013). Some studies found that work engagement can lead to well-being (Shimazu and Schaufeli, 2009; Schaufeli, 2012; Shimazu et al., 2012; Shuck and Reio, 2014) while other studies argued that well-being could also contribute to the work engagement (Upadyaya and Salmela-Aro, 2013). According to the broaden-and-build theory of positive emotions (Fredrickson, 2001), positive emotions share the capacity to broaden people's momentary thought-action repertories and can be transferred into decisions and actions. Happy people are found to be more sensitive to opportunities at work, urge to play, and be creative and have more desire to explore and assimilate new experiences, producing higher levels of vigor and absorptions at work (Cropanzano and Wright, 2001). Fredrickson and Losada (2005) showed that when managers' positive emotion level was high, they asked more questions in business meetings, their range between questioning and advocacy was broader, and they had more vigor and engagement in their work. As discussed above, we proposed that well-being can contribute to the work engagement, and well-being can be a potential mediator between growth mindset and work engagement.

### Perseverance of Effort as Mediator

Besides well-being, perseverance of effort could be a potential mediator in the relationship between growth mindset and work engagement. The relationship between growth mindset and perseverance of effort was found in different studies. For example, Puente-Diaz and Cavazos-Arroyo (2017) found the positive relationship between growth mindset and effort, and they explained that growth mindset is “related to a preference for progress cues emphasizing learning and improvement” (p. 4). Additionally, Zhao et al. (2018) found that growth mindset predicted grit, and internal motivation mediated this relationship among Chinese students. Grit was defined as “perseverance of effort and consistency of interest” (Suzuki et al., 2015, p. 1). Also, Burnette et al. (2013) meta-analysis demonstrated that implicit theories predicted the self-regulatory process, which in turn, predicted the perseverance of effort and goal achievement.

In terms of the relationship among growth mindset, perseverance of effort and engagement, Zeng et al. (2016) explained that students' growth mindset was positively related to school engagement because they saw effort as one of the effective approaches to improving ability, intelligence and experiences. Blackwell et al. (2007) also found that students with a growth mindset demonstrated more effort in studying because these students believed that their intellectual abilities could always be further developed through effort and hard work. The reason for the positive relationship between college students' work engagement and academic effort could be that students were more engaged in their academic work, felt better and then put more effort academically (Strauser et al., 2012). Individuals would be persistent on the tasks even when they encounter challenges and difficulties. The type of mindset predicted variables of goal setting (e.g., performance and learning goals), goal operation (e.g., helpless and mastery-oriented strategy), and goal monitoring (e.g., negative emotion and expectations), which contributed to the perseverance of effort and goal achievement (Leondari and Gialamas, 2002; Ahmavaara and Houston, 2007). Dupeyrat and Mariné (2005) studied a group of adults who returned to school for their high school diploma and found that adults with growth mindset showed more effort to work and avoided work less. On the contrary, when intelligence was thought to be static, mastery goals, which were found to significantly predict deep learning strategies and effort, were set less. Taken together, previous studies indicated that perseverance of effort might serve as a bridge and mediate the growth mindset on work engagement.

## The Chinese Educational System

In contrast to the numerous studies conducted in developed countries, very few studies on teachers' work engagement, work stress, and job satisfaction have been conducted in developing countries (Garrett, 1999; Zembylas and Papanastasiou, 2004). In China, this kind of research is still at an early stage (Sun and Pan, 2008; Liu and Onwuegbuzie, 2012). The limited studies have shown that many Chinese teachers have suffered from a high level of work stress, burnout and turnover rate (Chan and Hui, 1995; Chan, 1998; Hui, 1998; Liu and Onwuegbuzie, 2012). Qualitative data showed that approximately 40% of Chinese secondary teachers in the sample reported that they probably or certainly would leave the teaching profession due to high levels of stress, low salaries, inadequate breaks and holiday, heavy workload and student behaviors (Wong, 1989; Liu and Onwuegbuzie, 2012). A study found that the average working hours of Chinese secondary school teachers of the sample were almost 48 h per week and teach almost four classes per day (Zhang and Zhu, 2007). Teacher stress was found to have a strong effect on burnout in Chinese secondary education, and overload was the most common stressor of burnout (Chan, 1998; Hui, 1998; Zhang and Zhu, 2007). As a result, it is significant to explore the possible approaches to improve work engagement of Chinese teachers and reduce their burnout as well as work stress.

Furthermore, in contrast to the majority of growth mindset studies that have been conducted with Western samples (e.g., Dweck and Leggett, 1988; Good et al., 2003; Jonsson and Beach, 2012), very few studies have investigated growth mindset and work engagement in Eastern cultures, such as Chinese culture. It is essential and valuable to examine whether the findings of previous studies in Western culture can be generalized to other cultures as well. Up-to-date, the current study is the first to examine growth mindset and its impacts on well-being, perseverance of effort and work engagement of teachers in the Chinese educational system.

## The Present Study

**Figure 1:**
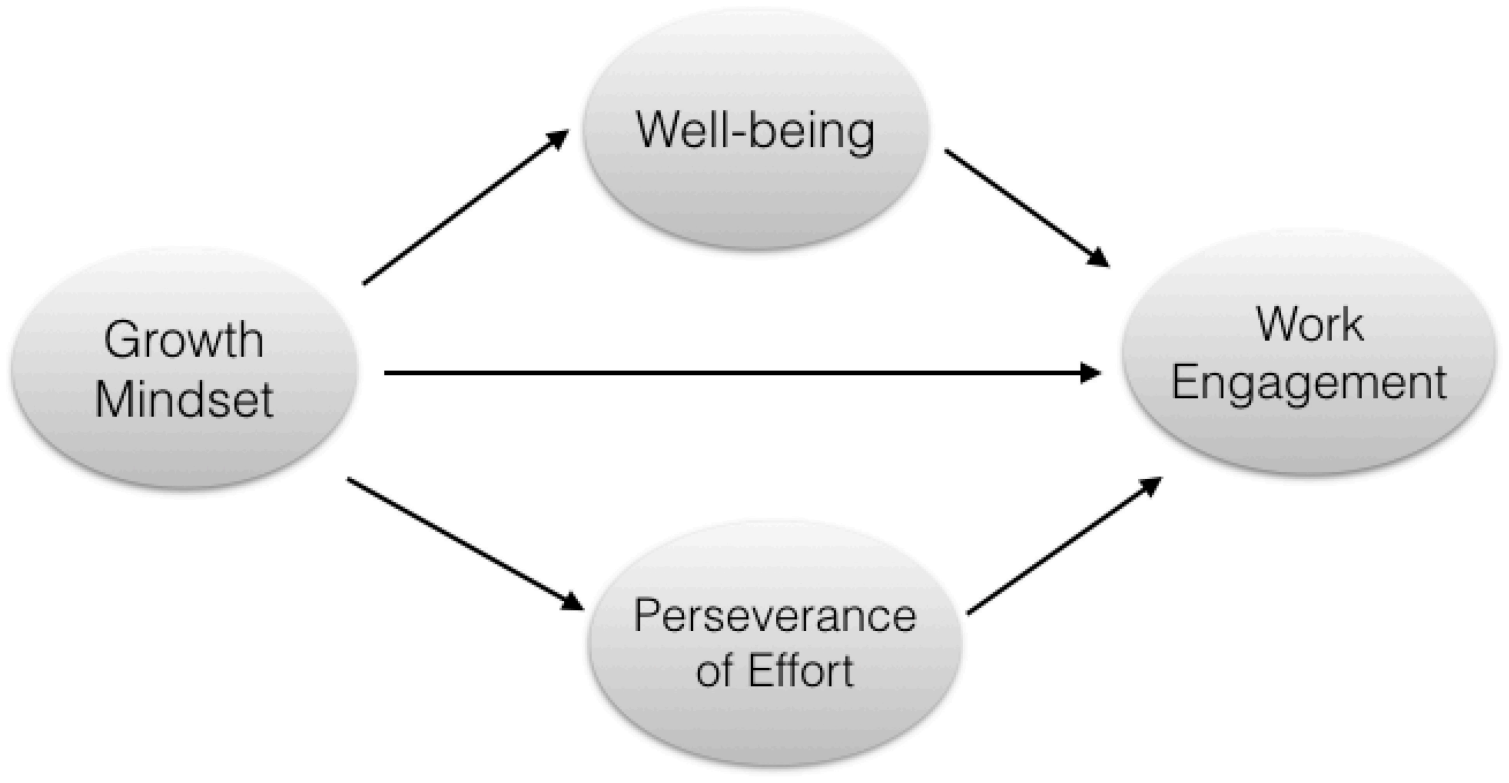
A conceptual model of relationship between Growth mindset, Well-being, Perseverance of effort, and Working engagement^1^.

This study intended to investigate the relationships amongst growth mindset, well-being, perseverance of effort, and work engagement in the Chinese educational system. As we discussed above, we proposed our conceptual model in Figure 1. Specifically, we hypothesized that: (1) growth mindset could positively predict work engagement directly; (2) well-being mediated the association between growth mindset and work engagement; and (3) perseverance of effort mediated the relationship between growth mindset and work engagement.

## Method

### Participants

Four hundred and seventy-two teachers were recruited from 10 secondary schools in Chengdu city, the capital city of the province of Sichuan in central China, to participate in this study and to complete the survey. In this sample, their ages ranged from 18 to 60 (*Mage* = 39.61, *SD* = 8.62); there were 331 (70.3 %) females and 140 (29.7%) males. There were 423 (89.6%) teachers from middle schools and 49 (10.4 %) of them were from high school. These 10 participating schools are located in the urban areas of Chengdu city. On average, 9% of teachers participated at each school site (participation ranged from 5 to 14% across all sites). The average number of participating teachers was 42 (ranging from 22 to 64 teachers).

### Measures

#### Work Engagement

The Chinese version of Utrecht Work Engagement Scale (UWES), which was originally developed by Schaufeli et al. (2006), was used to assess Chinese work engagement (Yi-wen and Yi-qun, 2005). The measure was demonstrated to be satisfactory reliability and validity (Yi-wen and Yi-qun, 2005; Fong and Ng, 2012). UWES includes three subscales: vigor (six items; e.g., “*At my job, I feel strong and vigorous”), dedication (*five items*;* e.g.*, “I find the work that I do full of meaning and purpose*.”) and absorption (six items; e.g., “*Time flies when I am working*.”). Responses were made on a 7-point Likert-type scale from 0 = (never) to 6 = (always). The Cronbach's alpha of this scale was 0.96 in this study (0.87 for vigor subscale, 0.89 for dedication subscale, 0.91 for absorption subscale).

#### Growth Mindset

The study administrated a Chinese version of 4-item Growth Mindset Inventory, which was originally developed by Dweck (2006) to measure the degree of the growth mindset of responders. The Chinese version of Growth Mindset Inventory was shown satisfactory reliability and validity (Zeng et al., 2016). Participants rated the items using a 5-point Likert scale from 1 = (*strongly disagree)* to 5 = (*strongly agree)*. An example item is such “*You can always substantially change how intelligent you are*.” In this study, Cronbach's alpha for the growth mindset subscale is 0.83.

#### Well-Being

In terms of Well-being, as defined by Seligman (2011), the overall well-being consists of five factors: positive emotion (three items; e.g.*, “*In general, how often do you feel joyful?”), engagement (three items; e.g., “*How often do you become absorbed in what you are doing?”*), relationship (three items; e.g.*, “To what extent do you receive help and support from others when you need it?”*), meaning (three items; e.g*., “In general, to what extent do you lead a purposeful and meaningful life?”*), and accomplishments (e.g., three items; e.g., “*How often do you achieve the important goals you have set for yourself?”*). We used the PERMA profiler (Butler and Kern, 2016) to measure five dimensions of Well-being. Responders rate themselves on a ten-point Liker scale 0 = (*never/very bad)* to 10 = (*always/very good)*. A Chinese version of PERMA scale was received from its developer, Margaret Kern. The Chinese version of PERMA scale was demonstrated to be satisfactory reliability and validity (Lai et al., 2018). In this study, Cronbach's alpha for the whole scale of well-being was 0.95. The Cronbach' alpha for the subscale of positive emotion, engagement, relationship, meaning and accomplishments are 0.89, 0.76, 0.77, 0.89, 0.81, respectively.

#### Perseverance of Effort

Perseverance of Effort was measured by using 4-item Effort subscale from Grit-Scale, which involves sustaining effort in the face of adversity. Response options ranged from 1 = (*not at all like me)* to 5 = (*very much like me*). Duckworth's laboratory translated the Chinese version of the Grit-S used in the present study. Li et al. (2016) used a back-translation procedure to ensure the accuracy of the original translation and reached the consensus that any additional changes were unnecessary. An example item was “*I am a hard worker*.” Cronbach's alpha of the whole scale in this study was 0.72.

### Procedures

This research received approval from the Human Research Ethics Committee of Tsinghua University. The research team contacted with the bureau of education in Chengdu city (Qingyang district) and 10 secondary schools volunteered to participate in this research project. One online survey link was sent to the school administrations of all the 10 participated schools. The teachers of the participated schools were all encouraged to participate in the study without compensation paid, and they answered the questionnaires through their mobile phone or computers. They completed the online questionnaires in the 2nd week of September 2017 (beginning of the semester). Ninety percent of participants completed the survey within 10 min. Informed consent was obtained from participants. Before the application of the questionnaires, the participants were informed about the objectives of this research project, and confirmed that all data would be kept confidential, only accessible to the research group and only can be used for research purposes.

## Results

### Descriptive and Preliminary Analysis

Correlations means and SDs for all study measures are presented in Table 1. As the table shows, the mean values of all the variables were on the higher side of the scale, and there was no significant gender and age impact on these variables. As shown in Table 1, all these variables were significantly correlated in an expected manner. Growth mindset was positively and strongly correlated with well-being (*r* = 0.59, *p* < 0.01), perseverance of effort (*r* = 0.63, *p* < 0.01) and work engagement (*r* = 0.70, *p* < 0.01). Moreover, both well-being and perseverance of effort were positively related to work engagement with values ranging from moderate to large (0.45 to 0.69).

**Table 1:** Descriptive analysis and correlations between all variables.

**Measures**	**1**	**2**	**3**	**4**
1. Growth Mindset				
2. Well-being	0.59 ^**^			
3. Perseverance of Effort	0.63^**^	0.45^**^		
4. Work Engagement	0.70^**^	0.69^**^	0.58^**^	
Range	1–5	0–10	1–5	1–6
*M*	3.29	6.07	3.61	3.76
*SD*	0.75	1.82	0.78	1.03

*N = 471*,

** *p < 0.01*.

### Structural Equation Modeling (SEM)

**Figure 2:**
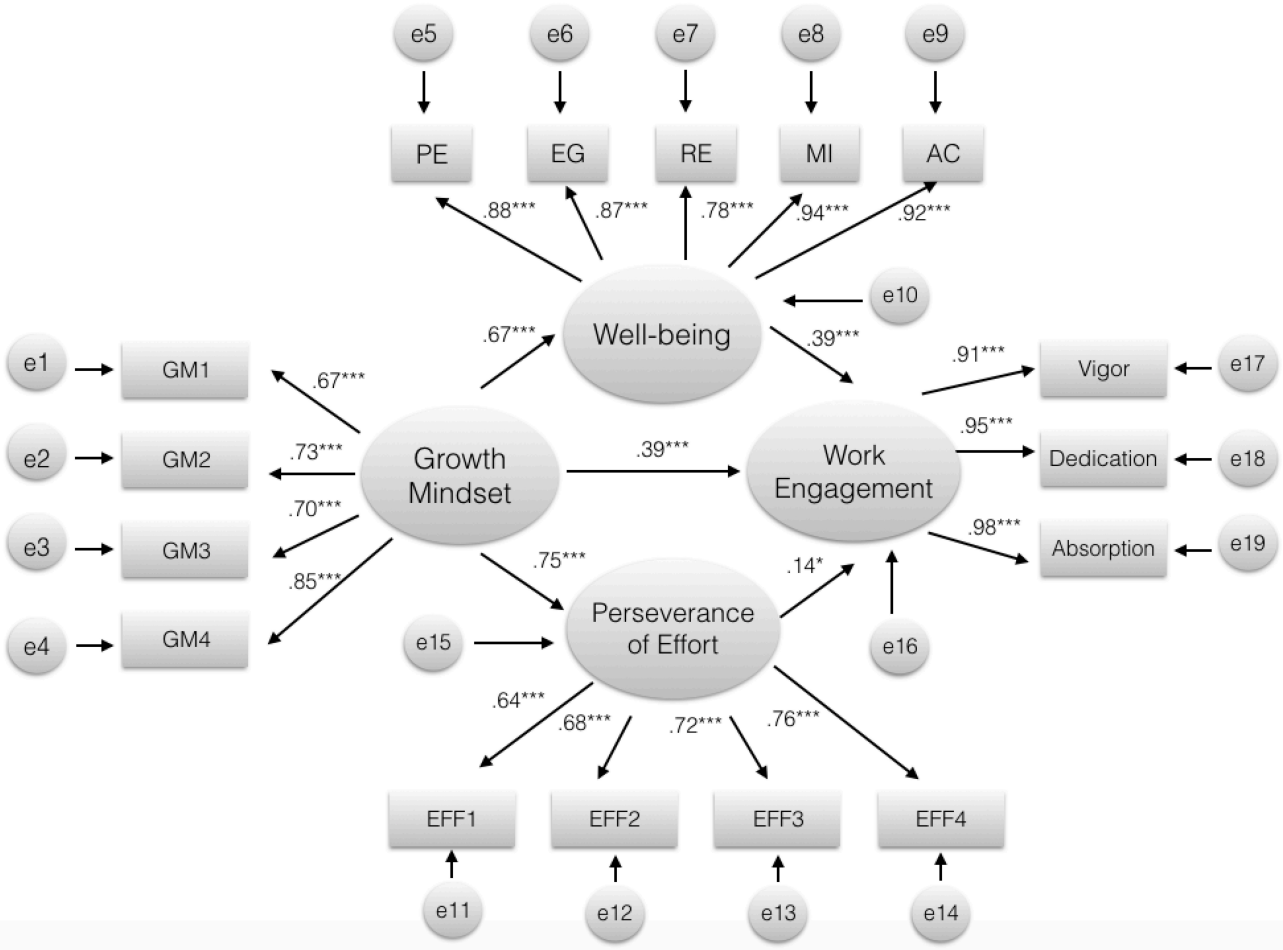
Estimated results of SEM (standardized estimates for statistically significant paths) for Growth mindset, Well-being, Perseverance of effort and Work engagement^1^.

Based on our conceptual model developed above, we used structural equation modeling (SEM) to examine the relationship between growth mindset, well-being, perseverance of effort, and work engagement (see Figure 1). A model consists the latent variables (growth mindset, well-being, perseverance of effort, and work engagement), and the observed variables (see the rectangles in Figure 2) of each latent factor (see the ovals in Figure 2). The SEM analysis was conducted with AMOS software. Next, we proceed to run a series of path analysis that started with our initial model. In the level of measurement models, all the loadings were significant, ranged from 0.64 to 0.98, suggesting that all the observed indicators could be explained by the latent variables. In terms of the path model, we referred to the comparative fit index (CFI), the Tucker-Lewis index (TLI), the root means square error of approximation (RMSEA), Standardized Root Mean Square Residual (SRMR) and Bayesian Information Criterion (BIC). CFI and TLI values >0.90 are considered to indicate acceptable model fit, and RMSEA and SRMR values at or below 0.08 indicate that the model provides a reasonable fit to the data (Hu and Bentler, 1998; Byrne, 2016). In this study, the resulting fit indices for this model were: χ2 = 414.689 (*df* = 99, *p* < 0.001), CFI = 0.95, TLI = 0.94, RMSEA = 0.08, SRMR = 0.056, BIC = 643.28, indicating a satisfactory fit to the data.

As shown in Figure 2, all proposed paths were significant at the 0.05 level or better. The paths from growth mindset to well-being (b = 1.61, *SE*. = 0.11, β = 0.67, *p* < 0.001) and to perseverance of effort (b = 0.74, *SE*. = 0.05, β = *0.7*5*, p* < 0.001), the path from well-being to work engagement (b = 0.21, *SE*. = 0.02, β = *0.3*9, *p* < 0.001), and the path from perseverance of effort to work engagement (b = 0.18, *SE*. = 0.07, β = *0.1*4, *p* < 0.05), were all significant and positive.

### Mediational Roles of Well-Being and Perseverance of Effort

The bootstrapping method with 10,000 bootstrap sample was used to test the indirect effects of the growth mindset on work engagement.

As shown in Table 2, results reported that after controlling for age (*p* = 0.98) and gender (*p* = 0.34), the paths from growth mindset to work engagement through well-being (0.26; *p* < 0.001; 95% CI:0.20 to 0.32) and through perseverance of effort (0.10; *p* < 0.00; 95% CI:0.05 to 0.16) were statistically significant, and the mediation effect was large, while the direct effect of growth mindset on work engagement was also statistically significant (0.36; *p* < 0.001; 95% CI:0.29 to 0.44). Taken together, the results indicated that the well-being and perseverance of effort partially mediated the association between growth mindset on work engagement.

**Table 2:** Standardized indirect effects of growth mindset on work engagement through well-being and perseverance of effort (controlling for gender and age).

			**95% BC** ***CI***	
**Mediator**	**Parameter estimate**	***SE***	**Lower**	**Upper**
**WORK ENGAGEMENT**				
Total	0.36	0.04	0.29	0.44^***^
Well-being	0.26	0.03	0.20	0.32^***^
Perseverance of Effort	0.10	0.03	0.05	0.16^***^

*N = 471. BC CI, bias- corrected confidence interval*.

*** *p < 0.01*.

In conclusion, these results in this study supported our conceptual model and suggested that the growth mindset could have both significant direct and indirect effect through well-being and perseverance of effort on work engagement.

## Discussion

With reference to the theoretical mid-point of the response scale (i.e., a score of 3 for a five-point Likert scale), the Chinese teachers perceived relatively high scores on growth mindset, well-being, and perseverance of effort, but relatively low scores on work engagement. The low work engagement could be due to the heavy daily workload, high sense of responsibility and stress in the Chinese educational context (Wong, 1989; Chan, 1998). Our results pointed out the need of promoting work engagement among Chinese teachers.

Correlation analysis showed that growth mindset, well-being, and perseverance of effort were positively correlated with work engagement, which supported our hypotheses. Moreover, consistent with our hypotheses, structural equation model further suggested that well-being and perseverance of effort could partially mediate the relationship between growth mindset and work engagement. The result demonstrated that having a growth mindset predicted the higher degree of well-being as well as perseverance of effort, which in turn positively influenced teachers' work engagement.

To the best of our knowledge, the full paths of the relationships among growth mindset, well-being, perseverance of effort, and work engagement in our mediating model have not been investigated before in other studies, especially within the Chinese educational system. However, several paths have been examined separately in previous literature. Our findings are consistent with previous studies on the following: (a) growth mindset predicts work engagement (Heslin, 2010; Keating and Heslin, 2015); (b) growth mindset predicts well-being (Zeng et al., 2016; Mouratidis et al., 2017; Whittington et al., 2017); (c) growth mindset is positively correlated with perseverance of effort (Strauser et al., 2012; Burnette et al., 2013; Zhao et al., 2018); and (d) well-being and work engagement is positively associated with each other (Shimazu and Schaufeli, 2009; Shimazu et al., 2015), and well-being could also contribute to promoting one's engagement level (Upadyaya and Salmela-Aro, 2013). Notably, previous studies showed that growth mindset could positively impact work engagement among employees in organizational settings (Heslin, 2010; Keating and Heslin, 2015) or students in schools (Blackwell et al., 2007; Zeng et al., 2016). Our results demonstrated that the prediction from growth mindset to work engagement was also established in the educational context among teachers, thus addressing its generalizability. Heslin (2010) explained that mindset could affect work engagement in several ways, such as zeal of development. For example, Heslin (2010) explained that students with a growth mindset could see taking courses, reading, or coaching as a valuable developmental opportunity for promoting self-growth.

### Explanation of Well-Being as a Mediator

As hypothesized, SEM analysis demonstrated that well-being functioned as a partial mediator between growth mindset and work engagement. Previous studies showed that the improvement of work engagement led to higher degree of well-being in the workplace (Shimazu and Schaufeli, 2009; Robertson and Cooper, 2010; Schaufeli, 2012; Shimazu et al., 2012; Shuck and Reio, 2014). The current study contributed to the literature by demonstrating that well-being could also contribute to work engagement, and the relationships between work engagement and well-being might be reciprocal.

Notably, we employed the PERMA model of well-being, proposed by Seligman (2011), which consisted of five dimensions: positive emotions, meaning, achievement, relationship, and engagement, to explain the relationship between well-being and work engagement. For example, the Broaden and Built theory maintained that the experience of positive emotions served to broaden the scope of people's attention, thought processes, and action; and in long-term, build physical, intellectual and social resources (Fredrickson, 2001; Fredrickson and Branigan, 2005; Cohn and Fredrickson, 2009), which in turn might contribute to the work engagement (i.e., vigor, dedication and absorption). A sense of meaning can give a direction and purpose to people's actions (Robertson and Cooper, 2010), and enhance their intention, motivation, and dedication to their work. Additionally, many studies also demonstrated that sense of achievement can effectively increase the motivation of employees (e.g., Dieleman et al., 2003; Franco et al., 2004), and therefore drove employees to be more engaged in their work. Additionally, interpersonal relationships can predict work engagement. For example, a study showed that interpersonal relationships with administrations were most predictive of managers' work engagement and proactive work behaviors (Warshawsky et al., 2012). However, it is important to note that the construct of well-being is complex, and previous studies measured well-being by employing various theoretical models. For example, Shuck and Reio (2014) operationalized well-being as a construct that includes four dimensions: emotional exhaustion, depersonalization, personal accomplishment, and psychological well-being. Robertson and Cooper (2010) represented well-being by psychological well-being, which consisted of positive relationships with others; personal mastery; autonomy; a feeling of purpose and meaning in life; and personal growth and development. Other studies defined well-being as being composed of high ill-health and low life satisfaction (Shimazu and Schaufeli, 2009; Shimazu et al., 2012). Some of the abovementioned well-being constructs are similar and overlapped with each other; therefore, future studies need to take into consideration of this issue.

### Explanation of Perseverance of Effort as a Mediator

Our results also showed that perseverance of effort served as a partial mediator between growth mindset and work engagement. In particular, perseverance of effort functioned as a mechanism to explain why teachers with growth mindset are high in work engagement. In views of effort, teachers with growth mindset or fixed mindset have very different perspectives. Teachers with growth mindset believe that effort to be essential in developing their intelligence and basic ability. Thus, they are motivated to invest effort for the tasks at hand to grow and develop. Additionally, when encountering challenges and difficulties, growth mindset individuals will show persistence on the tasks (Suzuki et al., 2015), be resilient (Zeng et al., 2016), and invest effort to solve them (Blackwell et al., 2007). In opposition, teachers with a fixed mindset hold the belief that basic ability and intelligence are the most essential elements for working, which cannot be altered and changed through effort and hard work. Fixed-mindset individuals believe that if one has the ability and intelligence, it is not necessary to expend considerable effort to achieve their goals. Therefore, fixed-mindset teachers are found to have a higher level of work avoidance (Burnette et al., 2013), tend to give up instead of persisting through difficulties (Dupeyrat and Mariné, 2005; Dweck, 2007), and avoid putting effort in various useful development opportunities.

In summary, the current study is the first to examine growth mindset and its influences on well-being, perseverance of effort, and work engagement of teachers in the Chinese educational background. Compared with the overwhelming amount of growth mindset research in Western cultures (Dweck and Leggett, 1988; Good et al., 2003; Jonsson and Beach, 2012), limited research has been conducted within Eastern culture. It is valuable to examine whether the results and conclusions can be generalized to Eastern cultures. Previous studies showed that Easterners and Westerners have different cognitive styles (analytic vs. holistic), social orientation (independence vs. interdependence), values (individualism vs. collectivism), and motivation (self-enhancement vs. self-criticism) (Markus and Kitayama, 1991; Morris and Peng, 1994; Peng and Nisbett, 1999; Nisbett et al., 2001). Our results confirmed that growth mindsets can predict well-being, perseverance of effort and work engagement in the Chinese culture.

This study suggested possible implications associated with the linkages among Chinese teachers' growth mindset, well-being, perseverance of effort and work engagement in school. The findings revealed that growth mindset and well-being of teachers are positively associated with their work engagement. Therefore, governments and school administrators may need to consider promoting teachers' work engagement in school through cultivating their growth mindset and well-being levels. Furthermore, perseverance of effort could partially mediate the relationships between growth mindset and work engagement. It might imply that school administrators could support teachers' perseverance of effort to strengthen the effects of growth mindset on work engagement.

### Limitations and Future Direction

In conclusion, the current study aimed to explore the direct and indirect predictive factors of work engagement of Chinese secondary school teachers. The findings suggested that growth mindset, well-being, and perseverance of effort could all predict work engagement. Moreover, well-being and perseverance of effort could serve as partial mediators between the growth mindset and work engagement. These findings pointed out the possible predictive factors in promoting the work engagement for Chinese secondary school teachers. It is important to find ways to build up teachers' growth mindset, well-being, and perseverance of effort, to make them feel more engaged into their work.

Some limitations in the current study should be acknowledged. First, this study was cross-sectional, the mediating model is insufficient in determining any causal relationships that may exist among growth mindset, well-being, perseverance of effort, and work engagement. Future studies should consider conducting experimental, prospective and longitudinal approaches to examine the causality among these variables. Moreover, the participants of the present study were recruited from mainland China, and the Chinese educational context may be different from other Asian countries and cultures. Future studies should consider expanding the samples to multiple cultures. Third, all data was based on teachers' self-evaluations, which could lead to common method variance concern. Employing multiple methods of assessment could be beneficial in future studies. Furthermore, in the current study, we focused on several factors to explain the mediating mechanisms of growth mindset and work engagement. However, there are also other factors that have important roles in influencing teachers' growth mindset and work engagement, such as self-efficacy (Strosher, 2003), resilience (Blackwell et al., 2007; Burnette et al., 2013; Zeng et al., 2016), and goal achievement orientation (Leondari and Gialamas, 2002; Ahmavaara and Houston, 2007). Therefore, further research needs to include these factors as mediating or moderating variable to better understand the relationship between growth mindset and work engagement.

## Ethics Statement

This study was carried out in accordance with the recommendations of Research Ethics Committee in psychology Department, Tsinghua university. The protocol was approved by the Research Ethics Committee in psychology Department, Tsinghua university. We used online questionnaires to invest teacher's psychological constructs, which has no harmful impacts on students. We also follow the informed consent discipline, which means that we told every Teacher's the information about this research, and invite them to participate in. If they do not want to participate, they can refuse to answer the questionnaires. Most of them agreed. No vulnerable populations were involved.

## Author Contributions

GZ: research design, data collection, substantial revision work. XC: research design, manuscript draft, and data interpretation, and corresponding author work. HC: research design, manuscript draft and interpretation. KP: research design, revision work.

### Conflict of Interest Statement

The authors declare that the research was conducted in the absence of any commercial or financial relationships that could be construed as a potential conflict of interest.

## Footnotes

^1^Well-being and Perseverance of effort are hypothesized to exert as uncorrelated parallel mediators between Growth mindset and Working engagement
